# 

**Published:** 1873-06-01

**Authors:** 


The next Annual Meeting of the North-Eastern Indiana Medical Society will be held at Kendallville, on Tuesday, June 3, 1873, at 10 o'clock a. m. Public address at 8 o'clock p. m., by T. A. McGraw, of Detroit.

J. L. GILBERT, Secretary.

